# Muscle-Tendon Interaction During Human Dolphin-Kick Swimming

**DOI:** 10.3389/fphys.2019.01153

**Published:** 2019-09-13

**Authors:** Kanae Sano, Takumi Sakamoto, Ryoma Nishimura, Yoshito Danno, Paavo V. Komi, Masaki Ishikawa

**Affiliations:** ^1^Faculty of Health Sciences, Morinomiya University of Medical Science, Osaka, Japan; ^2^Ritsumeikan Global Innovation Research Organization, Faculty of Sport and Health Science, Ritsumeikan University, Shiga, Japan; ^3^Department of Health and Sports Management, Osaka University of Health and Sport Sciences, Osaka, Japan; ^4^Likes Research Center, University of Jyväskylä, Jyväskylä, Finland

**Keywords:** ultrasonography, vastus lateralis, electromyography, muscle fascicle, swim

## Abstract

Without high impact forces, it is not clear how humans can utilize tendon elasticity during low-impact activities. The purpose of the present study was to examine the muscle-tendon behavior together with the electromyographic (EMG) activities of the vastus lateralis (VL) muscle during the human dolphin-kicking. In a swimming pool, each subject (*n* = 11) swam the 25 m dolphin-kicking at two different speeds (NORMAL and FAST). Surface EMGs were recorded from the VL and biceps femoris (BF) muscles. Simultaneous recordings of the knee joint angle by electro-goniometer and of the VL fascicle length by ultrasonography were used to calculate the muscle-tendon unit and tendinous length of VL (L_MTU_ and L_TT_, respectively). In the dolphin-kicking, the stretching and shortening amplitudes of VL L_MTU_ did not differ significantly between the two kicking speed conditions. However, both stretching and shortening amplitudes of the VL fascicle length were lower at FAST than at NORMAL speed whereas the opposite was found for the VL L_TT_ values. At FAST, the contribution of the VL tendinous length to the entire VL_MTU_ length changes increased. The EMG analysis revealed at FAST higher agonist VL activation from the late up-beat (MTU stretching) to the early down-beat phases as well as increased muscle co-activation of VL and BF muscles from the late down-beat to early up-beat phases of dolphin-kicking. These results suggest that at increasing kicking speeds, the VL fascicles and tendinous tissues during aquatic movements can utilize tendon elasticity in a similar way than in terrestrial forms of locomotion. However, these activation profiles of VL and BF muscles may differ from their activation pattern in terrestrial locomotion.

## Introduction

In terrestrial locomotion, the actively stretched skeletal muscle can enhance the power and/or efficiency, following the stretch-shortening cycle (SSC) concept (cf. Komi, 1992, 2003). In running and jumping, for example, impact forces can be converted and then be utilized as elastic energy during the ground contact phase. With higher running speeds and drop heights, the stored and released elastic strain energy can be increased within the muscle-tendon unit (MTU) (e.g., Bosco et al., 1981; Ishikawa and Komi, 2004, 2008). In aquatic movements, however, this is likely not the case given the low impact load and slow movement conditions due to hydrodynamics, viscosity, and buoyancy as compared to the movements on land (Grimston and Zernicke, 1993; Enoka, 2015). Therefore, it remains questionable whether humans can utilize elastic strain energy caused by muscle-tendon interactions to improve performance during relatively low impact movements such as in swimming. This information is important when considering the training strategies of elite swimmers.

For underwater animals such as dolphins and tunas, their muscles possess long tendons (Bennett et al., 1987; Shadwick et al., 1999; Alexander, 2002). However, other studies suggested that tendons may not play very important roles as energy-saving springs during swimming when they calculated the tendon compliance for fishes (Bennett et al., 1987; Alexander, 1997, 2002). Therefore, many animal studies of swimming mechanics and energetics have been focused on temporal relationship between the muscle activation and muscle strain cycle *in vivo* (Shadwick et al., 1999; Shadwick and Syme, 2008). Shadwick et al. (1999) used sonomicrometry and electromyography to record the length changes of muscle fascicles and muscle activity during the tuna swimming, and they also found that the onset of muscle shortening on each side of the body exactly coincides with the timing when the body bends toward that side. However, the inertial force (muscle activation) on the tail occurs prior to the muscle fascicle shortening at the extremes of its side to side movements (Shadwick and Syme, 2008). If these forces can stretch the tendon greatly during the stretching of entire muscle tendon unit, the elastic strain energy would be utilized during the tuna swimming.

It has not been investigated whether human muscle fascicles during swimming can behave similarly as those of underwater animals. In humans, there are many reports about muscle activities during swimming (Martens et al., 2015), but little has been reported on the muscle-tendon behavior during swimming together with muscle activities. Recent musculoskeletal ultrasonography make now possible to examine muscle fascicle behavior during dynamic human movements. The vastus lateralis muscle is one of the major muscles for knee extension-flexion movements and it is easily detectable during human dynamic movements. Therefore, the purpose of the present study was to examine the muscle-tendinous behavior together with muscle activities of VL during the human dolphin-kick of swimming at different kicking speeds. It is hypothesized that during the human dolphin-kicking, the muscle fascicles and tendon can perform a SSC action. At higher swimming speeds, the greater stretching and shortening of the vastus lateralis tendinous tissues should be observed during the human dolphin-kicking. Therefore, the tendon elasticity might play important roles during human movements not only on land but also under water conditions.

## Materials and Methods

### Subjects

Eleven competitive male swimmers (butterfly and backstroke specialists) volunteered to participate in the study (Table 1). All subjects participated in swimming activities 5–6 times a week. This study was carried out in accordance with the recommendations of research guideline, the human Ethics Committee of Osaka University of Health and Sport Sciences with written informed consent from all subjects. All subjects gave written informed consent in accordance with the Declaration of Helsinki. The protocol was approved by the human Ethics Committee of Osaka University of Health and Sport Sciences (authorization n°13-28).

**Table 1:** Physical characteristics of the subjects (mean ± SD).

**Swimmer (*n* = 11)**	
Age (years)	20.9 ± 1.5
Height (m)	1.72 ± 0.06
Body mass (kg)	66.4 ± 4.7
Thigh length (m)	0.376 ± 0.034
VL MTU length (m)	0.372 ± 0.034
VL tendinous tissue length (m)	0.306 ± 0.036
VL fascicle length (mm)	70.3 ± 6.9

### Protocols

In an outdoor swimming pool (25 m), each subject was requested to swim 25 m underwater using the dolphin-kick (legs only) without breathing. After warming-up, the following devices were attached on the right leg: the surface electrodes for recording thigh electromyographic activities (EMG), a goniometer (SG150/W, Biometrics Ltd., UK) for the knee joint angle and an ultrasound probe (Prosound α10, Hitachi-Aloka Inc., Japan) for the VL muscle length changes. After preparation of the measurements, the subjects swam the dolphin-kicking at two different speeds (Supplementary Material). First, each subject was requested to swim the 25 m dolphin-kicking with maximum effort (FAST). After sufficient rest for about 10 min, each subject was requested to swim the submaximal 25 m dolphin-kicking (NORMAL: at 60% of the maximum dolphin-kicking speed). In this NORMAL condition, they were given a 25 m target time based on their 25 m dolphin-kicking with maximal effort. After measurements of dolphin-kicking, each subject performed isometric maximum voluntary contraction (MVC) measurements. MVCs for knee extension and flexion were performed twice, to replicate the amount of knee extension and flexion assumed during dolphin-kicking (at a joint angle corresponding to ~60° of knee flexion). The greater MVC trial was adopted to normalize muscle activities of BF and VL to MVC, respectively.

### Measurement Parameters

#### The Surface Electromyographic (EMG) Activity and Knee Joint Angle Recordings

Muscle activities were recorded from the VL and biceps femoris (BF) muscles of the right leg using bipolar surface electrodes (diameter 6 mm, inter-electrode distance 20 mm; Blue Sensor N-00-S/25, Ambu Medicotest A/S, Olstykke, Denmark) with a multi-telemeter system (sampling frequency 2 kHz, input impedance >10 MΩ, common mode rejection ratio >80 dB, time constant: 0.03 s; P-EMG plus, Oisaka electronic equipment, Japan). The electrode placements followed the SENIAM (Surface ElectroMyoGraphy for the Non-Invasive Assessment of Muscles) guidelines (Hermens et al., 2000) as accurately as possible. The VL electrodes were placed slightly lateral to the muscle mid-belly to accommodate the ultrasound probe. Before electrode placement, the skin was cleaned with alcohol. EMG signals were amplified (×1,000). The knee joint angular movements were recorded using a flexible electro-goniometer (SG150/W, Biometrics Ltd., UK). The flexible electro-goniometer was mounted along the line from the tip of the trochanter to the knee rotation center and the line from the knee rotation center to the lateral malleolus tip to measure knee joint angles during dolphin-kicking. The EMG signals and joint angle data were stored simultaneously to a personal computer through an analog digital converter with a sampling frequency of 2 kHz (Power 1401; Cambridge Electronics Design Limited, Cambridge, UK).

#### Ultrasonography Recordings

Ultrasonography was applied to record the longitudinal images of the VL fascicle length in the upright position and during dolphin-kicking. An ultrasound probe, which weigh ~130 g, was positioned over the VL muscle belly of the right leg to measure the fascicle length and pennation angle in the upright position as well as during the dolphin-kicking. The ultrasound probe with a 6 cm long linear array probe (scanning frequency: 13 MHz, image frequency: 117 Hz, Prosound α10, Hitachi-Aloka Inc., Japan) was used for all subjects. Similar to prior investigations during locomotion (Finni et al., 2001a,b; Kawakami et al., 2002; Ishikawa et al., 2003; Ishikawa and Komi, 2004), the ultrasound probe was positioned over the mid-belly (Blazevich et al., 2006) of the VL muscle and secured with a custom-made Styrofoam cast and wrapped tightly around the thigh to minimize any probe movement during the dolphin-kicking.

An electronic pulse was used to synchronize the EMGs, knee joint angle data and ultrasound data. At each kicking speed, 8–10 successive dolphin-kick cycles of the right leg were recorded once the swimming pattern was stabilized.

### Data Analyses

One cycle of the dolphin-kicking includes two phases based on knee joint movement; the up- and down-beat kick phases. The up-beat and down-beat kick phases were determined by the transition point from the flexion and extension of the knee joint (**Figure 2**). Moreover, the up-beat and down-beat kick phases were divided equally into the early and late parts. From the 8–10 recorded dolphin-kick cycles, all of them were kept for the knee joint angle and EMG analyses whereas the ultrasonography analyses concentrated on 3 successive dolphin-kick cycles. The knee joint angle data was filtered with a Butterworth fourth-order filter (cut-off frequency 10 Hz). The obtained knee joint angles were used to calculate the instantaneous VL muscle-tendon unit length (VL L_MTU_) with the model of Visser et al. (1990). The superior and inferior aponeuroses as well as a VL fascicle was identified and then digitized from each ultrasonographic image. The instantaneous length of VL tendinous tissues (VL L_TT_), which was defined as the sum of the proximal and distal tendinous structures and aponeuroses, was calculated by subtracting VL fascicle length multiplied by the cosine of its pennation angle from L_MTU_ (e.g., Finni et al., 2000, 2001a,b, 2003; Ishikawa et al., 2003):

(1) $$\begin{array}{r} \text{VL }{\text{L}}_{\text{TT}}=\text{VL }{\text{L}}_{\text{MTU}}\;-\text{ VL fascicLe Length }∙\text{ cos }\theta \end{array}$$

where VL L_TT_ is the instantaneous length of VL tendinous tissues, VL L_MTU_ is the instantaneous VL muscle-tendon unit length, and θ is the VL pennation angle created by the VL fascicle line and its attachments on the aponeurosis lines. This allowed the calculation of their corresponding mean knee angular change as well as amplitudes of VL L_MTU_, L_TT_, and fascicle length changes. The reliability and reproducibility of the ultrasound method of fascicle length calculation has been reported in previous studies (Kawakami et al., 2002; Ishikawa et al., 2003; Giannakou et al., 2011). The normalized two-dimensional cross-correlation coefficient was used to show the reproducibility of the ultrasound images during the 1 cycle of dolphin-kicking between 2 cycles for each subject. The correlation coefficient for images during 1 cycle of dolphin-kicking was on an average 0.918 ± 0.001. The reliability of the ultrasound method of calculating the VL fascicle length was determined by calculating the coefficient of variation between each point during the dolphin-kicking. The mean value was 4.5 ± 0.3%.

The EMG signals were band-pass fourth-order filtered (25–450 Hz) and full wave rectified. These signals and joint angular data of 8–10 stable cycle kicks were averaged for each cycle to get the averaged time course data for each subject whereas the ultrasonography analyses concentrated on three successive cycles. Thereafter, the averaged EMG amplitudes (aEMG) were calculated individually and separately for the four different phases during the dolphin-kicking (see above definitions). The recorded EMG values for VL and BF muscles during swimming were, respectively, normalized relative to knee extension/flexion MVC EMG values for each subject to consider the muscle activation level for swimming.

### Statistics

All values are presented as means ± standard deviations in the text and figures. Statistical analyses were performed using paired *t*-test for comparing the stretching and shortening amplitude of the VL LMTU, fascicle length and LTT between NORMAL and FAST conditions. For EMG data, normalized average EMG amplitudes were compared using two-way repeated measures ANOVA and Bonferroni *post-hoc* tests when applicable were used to examine the main effects of each parameter and interaction between the conditions (NORMAL and FAST) and phases (early and late up-beat kick phases, early and late down-beat kick phases). A criterion alpha level of *P* < 0.05 was used to determine statistical significance for all data. SPSS 23.0 software was used for statistical analyses.

## Results

The actual mean dolphin-kick speeds were 1.3 ± 0.1 m s^−1^ at FAST and 0.9 ± 0.1 m s^−1^ at NORMAL. The 1 cycle times of the dolphin-kicking for NORMAL and FAST were 647 ± 104 and 468 ± 46 ms, respectively. The separated phase times during the up-beat kick phase were 370 ± 76 and 235 ± 23 ms, respectively, and those during the down-beat kick phase were 278 ± 32 and 233 ± 31 ms, respectively. These times (1 cycle, up-beat and down-beat kick phases) were significantly shorter at FAST than at NORMAL (*p* < 0.01, respectively). The transition timing from the up-beat to down-beat kick phases of the 1 cycle dolphin-kicking was significantly shifted earlier in FAST (50.3 ± 2.9%) than in NORMAL (56.7 ± 3.3%) (*p* < 0.001).

**Figure 1:**
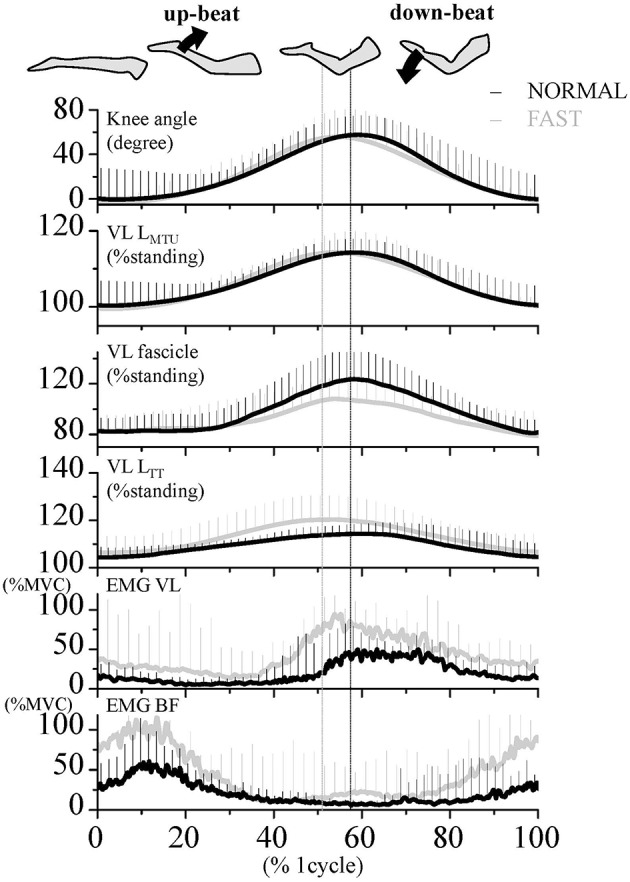
Time course of the knee joint angle, length of muscle-tendon unit (L_MTU_), fascicle and tendinous tissue (L_TT_) of the vastus lateralis (VL) muscle, as well as EMG activities of VL and biceps femoris (BF) muscles during dolphin-kicking at the NORMAL (Black line) and FAST (Gray line) speeds. These lines are the group mean data over the entire 8 cycles of joint angles and EMG and the 3 cycles of length change data, respectively. Vertical error bars on the curves indicate standard deviation (+1 SD). The mean EMG values were expressed relatively to the EMG amplitudes of the maximum voluntary contraction (MVC). The vertical lines show the maximal point of knee flexion angle during the dolphin-kicking in both conditions. VL L_MTU_, length of muscle-tendon unit; VL L_TT_, length of VL tendinous tissues.

**Figure 2:**
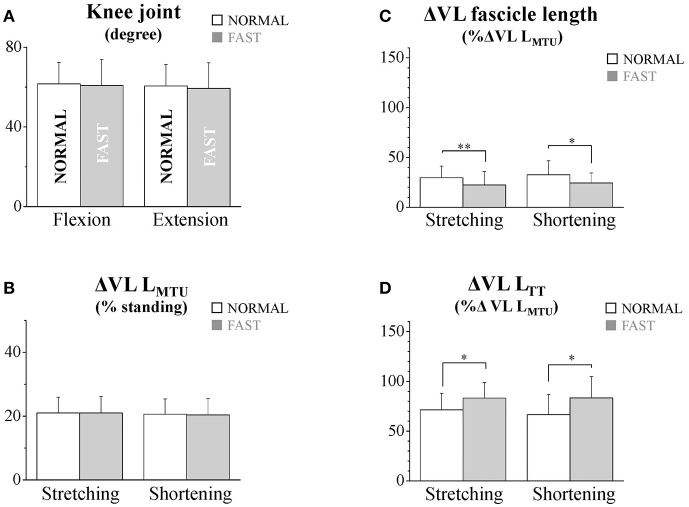
The flexion and extension amplitudes of the knee joint **(A)**, the stretching and shortening amplitudes of the vastus lateralis (VL) muscle-tendon unit **(B)**, fascicles **(C)**, and tendinous tissues **(D)** at NORMAL and FAST speeds during the dolphin-kicking. The knee flexion and extension amplitudes were calculated from the maximal knee joint extension to the maximal flexion (up-beat) and from the maximal knee joint flexion to that maximal extension (down-beat) during the dolphin-kicking. The stretching (up-beat kicking) and shortening (down-beat kicking) amplitudes were calculated at NORMAL and FAST, respectively. The error bars show the standard deviations for all subjects (+1 SD). ^*^ and ^**^ indicate significant differences between the NORMAL and FAST speed conditions at *p* < 0.05 and *p* < 0.01, respectively.

**Figure 3:**
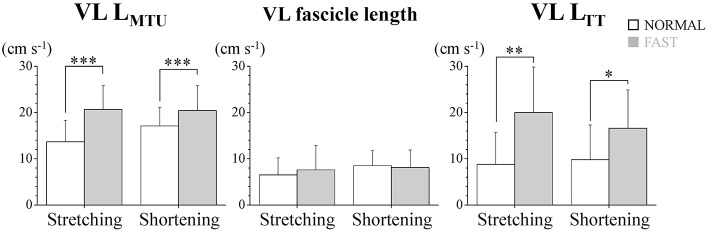
Stretching and shortening speeds of the muscle-tendon unit (VL L_MTU_), fascicle and tendinous tissue (VL L_TT_) of the vastus lateralis muscle (VL) during dolphin-kicking. The error bars show the standard deviations for all subjects (+1 SD). ^*^, ^**^, and ^***^ indicate significant differences between the NORMAL and FAST speed conditions at *p* < 0.05, *p* < 0.01, and *p* < 0.001, respectively.

During the dolphin-kicking, the amount of knee joint angular changes did not show any significant differences between both kicking speed conditions (Figures 1, 2A). In the length behavior, the VL fascicle length and VL L_TT_ as well as VL L_MTU_ at both NORMAL and FAST during the dolphin-kicking underwent a stretching and shortening behavior from the up-beat to down-beat kicking (Figure 1). The stretching and shortening amplitudes of the VL L_MTU_ did not show any significant differences between NORMAL and FAST (Figure 2B). However, the relative stretching and shortening amplitudes of the VL fascicle length were smaller at FAST than at NORMAL (ΔVL fascicle stretching: *p* < 0.01, ΔVL fascicle shortening: *p* < 0.05, respectively) (Figure 2C). Inversely, the stretching and shortening amplitudes of the VL L_TT_ were greater at FAST than at NORMAL speed (ΔVL L_TT_ stretching and shortening: *p* < 0.05, respectively). Although the stretching and shortening speeds of the VL fascicle length did not show any significant differences between NORMAL and FAST, the stretching and shortening speeds of the VL L_TT_ were faster at FAST than at NORMAL (ΔVL L_TT_ stretching: *p* < 0.01, ΔVL L_TT_ shortening: *p* < 0.05, respectively, Figure 3).

**Figure 4:**
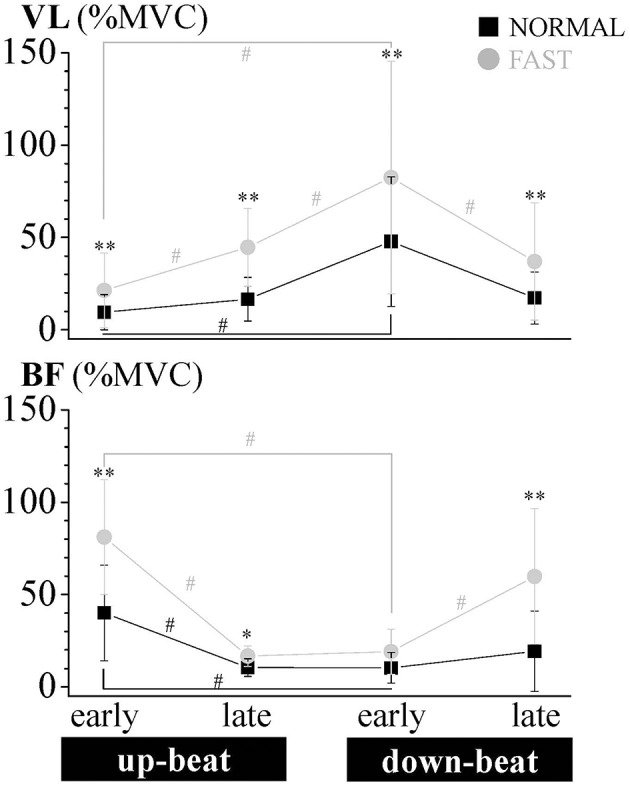
Relative aEMG comparison of the vastus lateralis (VL) and biceps femoris (BF) muscles for each phase of the dolphin-kicking between the NORMAL and FAST speed conditions. The aEMG of the 8 cycle data for each subject were averaged for each phase. The aEMG values were expressed as the relative values to the maximum voluntary contraction (MVC) values. The error bars show the standard deviations (± 1 SD) for all subjects. ^**^ and ^*^ show significant differences between conditions at *p* < 0.01 and *p* < 0.05, respectively. # shows significant differences between phases at *p* < 0.05.

The VL EMG activity at NORMAL increased gradually during the late up-beat kick phase and then reached its maximal muscle activation during the early down-beat kick phase (Figures 1, 4). On the other hand, the antagonist BF EMG activity was the highest at NORMAL speed during the early up-beat kick phase (Figures 1, 4). At increasing kicking speeds, the VL aEMG activity of each phase was significantly greater at FAST than at NORMAL (Figure 4). The BF aEMG activity during the early and late up-beat as well as the late down-beat kick phases were also significantly higher at FAST than at NORMAL.

## Discussion

The results of the present study clearly show that the VL muscle fascicles and tendinous tissues as well as the MTU present a SSC behavior during the knee joint flexion and extension during human dolphin-kicking. At faster speeds of the dolphin-kicking, the stretching and shortening amplitudes of the VL fascicle length were decreased and inversely, those of the VL L_TT_ were increased. The contribution of VL tendinous tissues to the entire VL MTU length changes increased at the faster speed of the dolphin-kicking. Therefore, not only on land but also under water, the efficient muscle force output prevents the increasing speeds of the muscle fascicle stretching and shortening. The increased utilization of the VL tendinous elasticity is considered as the common muscle-tendon strategy to enhance power output for faster human movements, such as running, jumping, and swimming. In the muscle activation patterns, however, not only the VL activation from the late up-beat (MTU stretching) to the early down-beat kick phases, but also specific muscle co-activation between VL and BF from the late down-beat to early up-beat kick phases of dolphin-kicking may play important roles to enhance kicking speeds.

### SSC Type of Muscle-Tendon Behavior During Human Dolphin-Kicking

In previous animal studies, the muscle stretching was calculated from body kinematics and/or EMG patterns during aquatic movements (e.g., Hess and Videler, 1984; Rome et al., 1993; Katz and Shadwick, 1998). They presumed that muscles perform predominantly positive work in animal swimming. However, such muscle work behavior during swimming would not necessarily be efficient. In human dolphin-kicking, the stretching and shortening behaviors of the VL muscle fascicles, tendinous tissues and MTU were observed in the same manner to running and jumping (Finni et al., 2001a,b; Ishikawa et al., 2003, 2005). At increasing kicking speeds, the stretching and shortening speeds of VL L_MTU_ and L_TT_ increased with the increased L_TT_ stretching and shortening amplitudes, although the speeds of VL fascicle length were unchanged and the amplitudes of VL fascicle length were decreased. These muscle fascicle and tendinous tissue interactions for enhancing performance of swimming are in line with those of previous studies in terrestrial locomotion (e.g., Ishikawa and Komi, 2008). Therefore, even in aquatic movements, the utilization of the VL tendinous elasticity would play an important role to enhance performance during the human dolphin-kicking. These may also explain the elastic behavior of connective tissues during the tuna swimming (Shadwick and Syme, 2008).

### Specific Muscle Behavior and Activation Profiles During Human Dolphin-Kicking

There is still a question as to whether the behavior of the MTU during low impact load swimming is the same as in high impact load jumps on land. Following the SSC concept (e.g., Komi, 1992, 2000), the pre-programed and stretch reflex muscle activation of agonist muscles is considered as playing important roles for performance enhancements through neuromuscular potentiation and utilization of elastic energy in drop jumps and hopping.

At both NORMAL and FAST speeds, not only VL but also BF was activated during the late down-beat phase in FAST. This BF antagonist muscle action (eccentric) can not only decelerate the knee extension at the end of the range of motion (ROM) but also enhance the kicking movements of the leg segment, causing the hyperextension of the swimmer knee. In the following early up-beat phase, both VL and BF were additionally activated at faster speed. BF then functions as an agonist (concentrically) to initiate knee flexion and inversely VL functions as an antagonist (eccentrically) to prevent knee flexion and fascicle lengthening. This increased antagonist VL activation concomitantly with the increased BF activation during the knee flexion at FAST can result in an increased muscle stiffness and function as the preprogramed muscle activation in SSC for complementing the low impact load dolphin kicking. On the other hand, a very small amount of co-activation occurred during the late up-beat and early down-beat phases. These different activation profiles among phases may be related to the gravitational loading and dominant kicking direction. Such co-activation during the transition phase has been observed in animal swimming (e.g., Altringham and Elleby, 1999), where the eccentric activation of an antagonist muscle results in an increased muscle stiffness and storage of elastic energy that serves to decelerate the joint at the end of the ROM. In the up-beat phase, the knee flexion and MTU stretching occurred gradually together with the BF and VL muscle activations at the early up-beat phase at FAST. In the following late up-beat phase, VL but not BF was highly activated leading to less VL fascicle lengthening as an eccentric action to decelerate knee flexion and to increase the stretching of the VL tendinous tissues efficiently in a similar fashion to the terrestrial locomotion (e.g., Ishikawa and Komi, 2008). Finally, during the early down-beat phase, VL continued to be active but functioned then as an agonist to initiate the knee extension. The reduced shortening velocity of VL muscle fascicles at FAST during the down-beat phase may have the advantage to produce force and power at the MTU level during the down-beat phase (VL shortening phase).

Thus, our results clearly showed that the VL stretching of the muscle fascicles and tendinous tissues did not start from the early up-beat but from the late up-beat kick phases in both conditions, increasing the contribution of VL tendinous tissues to the entire VL MTU length changes to enhance the kicking speed. However, at the low impact load swimming, not only the VL activation during the late up-beat phase but also the co-activated VL and BF muscles during the transition from the down-beat to up-beat phases can play important roles to compensate for the low impact loads and knee joint stiffness level due to hydrodynamics during swimming. Therefore, to enhance the power output of the MTU shortening during swimming, the high joint stiffness due to the co-activation from the late down-beat phase and high VL eccentric activation during the late up-beat phase can stretch the VL tendinous tissues effectively to enhance the kicking speed, even when the MTU stretching amplitudes were same in both conditions.

### Methodological Limitations

There are certain points in the methods that need to be addressed. The present study is perhaps the first one in which fascicle length (VL) and EMG activities were recorded simultaneously during human underwater swimming. We have measured only one knee extensor and flexor muscles during dolphin-kicking. However, other muscles at the knee, ankle, and hip joints can also contribute to dolphin-kicking and should be measured in the future studies. Secondly, the attached wired ultrasound probe and goniometers in the present study may influence the dolphin-kicking speeds. However, the maximal kicking speed value (1.3 ± 0.1 m s-1) for the FAST condition was similar in the speeds previously reported for male and female national level swimmers (1.2 ± 0.1 m s^−1^) (Connaboy et al., 2016) and for Olympian swimmers (1.5 m s^−1^) (Arellano et al., 2002; Loebbecke et al., 2009). The dolphin-kicking speeds in the FAST condition could be still high enough to examine the dolphin-kicking movements competitive swimmers.

## Conclusion

During human dolphin-kicking, the VL fascicles and tendon present a stretch-shortening action behavior. To enhance the swimming speeds of dolphin-kick, the tendinous elastic energy can play important roles for an efficient MTU force output. With faster swimming speed, the VL tendinous elastic energy was stored more from the late up-beat to early down-beat phases of dolphin-kicking. Even in this aquatic condition, a unique muscle co-activation of VL and BF muscles from the late down-beat to early up-beat phases of dolphin-kicking can keep and enhance the high VL muscle force and stiffness level during the up-beat phase of dolphin-kicking.

## Data Availability

All datasets generated for this study are included in the manuscript and/or the Supplementary Files.

## Ethics Statement

This study was carried out in accordance with the recommendations of research guideline, the human Ethics Committee of Osaka University of Health and Sport Sciences with written informed consent from all subjects. All subjects gave written informed consent in accordance with the Declaration of Helsinki. The protocol was approved by the human Ethics Committee of Osaka University of Health and Sport Sciences (authorization n°13-28).

## Author Contributions

KS, MI, and PK designed the experiment and wrote the manuscript. KS, TS, YD, and RN verified the analytical methods, performed the analyses, and interpreted the results. All authors performed the experiments, discussed the results, and commented on the final manuscript.

### Conflict of Interest Statement

The authors declare that the research was conducted in the absence of any commercial or financial relationships that could be construed as a potential conflict of interest.
